# Evaluation of Neonatal Streptozotocin Induced Diabetic Rat Model for the Development of Cataract

**DOI:** 10.1155/2014/463264

**Published:** 2014-11-19

**Authors:** Madhoosudan A. Patil, Palla Suryanarayana, Uday Kumar Putcha, Myadara Srinivas, G. Bhanuprakash Reddy

**Affiliations:** ^1^Department of Biochemistry, National Institute of Nutrition, Jamai-Osmania, Hyderabad 500007, India; ^2^Department of Pathology, National Institute of Nutrition, Hyderabad 500007, India

## Abstract

Type 2 diabetes (T2D) generally follows prediabetes (PD) conditions such as impaired fasting glucose (IFG) and/or impaired glucose tolerance (IGT). Although studies reported an association of IGT or IFG with cataract, the experimental basis for PD associated cataract is not known. Hence, we evaluated neonatal streptozotocin (nSTZ) induced rat model to study PD associated cataractogenesis by injecting STZ to two-day old rats. While majority (70%) of nSTZ injected pups developed IGT (nSTZ-PD) by two months but not cataract even after seven months, remaining (30%) nSTZ rats developed hyperglycemia (nSTZ-D) by two months and mature cataract by seven months. Lens biochemical analysis indicated increased oxidative stress as indicated by increased SOD activity, lipid peroxidation, and protein carbonyl levels in nSTZ-D cataractous lens. There was also increased polyol pathway as assessed by aldose reductase activity and sorbitol levels. Though nSTZ-PD animals have not shown any signs of lenticular opacity, insolubilization of proteins along with enhanced polyol pathway was observed in the lens. Further there was increased oxidative stress in lens of IGT animals. These results suggest that oxidative stress along with increased polyol pathway might play a role in IGT-associated lens abnormalities. In conclusion, nSTZ-PD rat model could aid to investigate IGT-associated lens abnormalities.

## 1. Introduction

Diabetes mellitus (DM) is a group of metabolic disorders characterized by circulating hyperglycemia. Type 2 diabetes is more prevalent in humans and accounts for 90–95% of all diabetic cases worldwide [1]. It is caused by a combination of resistance to insulin action and impaired insulin secretion [2]. The prevalence of diabetes for all age groups worldwide was found to be 382 million which may rise to 592 million by 2035 [3], whereas in India the current prevalence of diabetes is 40.9 million and is expected to rise to 69.9 million by 2025 [4–6]. Without proper management diabetes can lead to various complications such as cataract, retinopathy, nephropathy, neuropathy, and cardiovascular problems. Cataract, characterized by the opacification of the eye lens that interferes with transmission of light onto the retina, is one of the earliest secondary complications of diabetes. Cataract is the leading cause of visual impairment across the world and accounts for 47.8% of the blind people in the world [7]. Studies indicate that the incidence of cataract is much higher in diabetic than in nondiabetic individuals [8, 9].

Though the etiology of cataract is not fully understood, oxidative damage to the constituents of the eye lens is considered to be a major mechanism in the initiation and progression of various types of cataracts, including diabetic cataract [10]. Many studies indicate the role of oxidative stress in the development of diabetic complications including cataract due to glucose autoxidation, nonenzymatic glycation of proteins leading to advanced glycation end products (AGE), and enhanced glucose flux through polyol pathway [11–13]. More recent studies indicate that the polyol pathway is likely a major contributor to oxidative stress, at least, in the lenses and nerves of diabetic mice and it was reported that aldose reductase inhibitor reduces oxidative stress enhancement in sugar cataract [14]. The polyol pathway may be related to hyperglycemia induced oxidative stress, and there may be a metabolic connection between the polyol pathway and oxidative stress. Furthermore, carbonyl stress has been found to be the cause for advanced glycation end product toxicity indicating the involvement of nonenzymatic glycation in the development of oxidative stress.

Type 2 diabetes (T2D) always precedes with prediabetic conditions such as impaired glucose tolerance (IGT) or impaired fasting glucose (IFG) [6]. Increased oxidative stress was found in subjects with IGT and IFG [15]. Interestingly, several recent large population studies reported that IGT or prediabetes is also associated with various types of cataracts [16, 17]. There are three principle mechanisms through which lens can be damaged resulting in the development of cataract (oxidative stress, osmotic stress, and nonenzymatic glycation) and IGT might influence any or all of these physiological processes. However, no experimental studies have explained the association between IGT and cataract along with their plausible pathophysiological mechanisms. Previous studies from our group have shown that obesity [18, 19] as well as prediabetes [20] is associated with increased susceptibility to cataract formation due to the activation of sorbitol pathway in experimental animals. Thus, there is a need to understand the mechanism of cataractogenesis during IGT as well as T2D. In this context, availability of a suitable animal model that shows increased susceptibility to IGT-induced cataractogenesis is of immense value.

There are several animal models available to study the mechanisms of diabetic complications including diabetic cataract. However, most of the studies on diabetic cataract in animal models are largely restricted to type 1 diabetic (T1D) conditions [21–23]. In this context streptozotocin (STZ) [21, 24, 25] or alloxan-induced diabetic models [26–28] are extensively used to study the diabetic cataract, and both these models mimic the T1D in humans. Studies from our laboratory and elsewhere indicate that oxidative stress appears to be a major factor in the development of cataract in T1D, along with the activation of polyol pathway and nonenzymatic glycation [21, 22, 29].

Neonatal-STZ (nSTZ) induced rat model is one of the most frequently used T2D-like models [30–33] which mimics human diabetes [34]. Previous studies on this model were found to have characteristics of pancreatic beta-cell destruction followed by beta-cell regeneration and glucose intolerance [35, 36]. Subsequently, other authors confirmed these findings and showed that nSTZ treated rats in adulthood display the typical characteristics of T2D [37–40]. This animal model is mainly used to screen hypoglycemic or antidiabetic agents [32, 41, 42] and also for hypolipidemic and oxidative stress related studies [43]. However, no studies attempted to use this model for investigating the biochemical abnormalities in the lens and development of cataract. Therefore, in the present study we evaluated nSTZ model for development of cataract due to IGT or T2D and associated biochemical alterations in lens in rodent model.

## 2. Materials and Methods

### 2.1. Materials

Streptozotocin (STZ), NADPH, DL-glyceraldehyde, lithium sulfate, *β*-mercaptoethanol, bovine serum albumin, sorbitol and sorbitol dehydrogenase, tetraethoxypropane, thiobarbituric acid, NAD, and pyrogallol were purchased from Sigma Chemical Company (St. Louis, USA). All other chemicals were of analytical grade and were obtained from local companies.

### 2.2. Experimental Design

Two-day-old male Sprague Dawley (SD) rat pups obtained from the National Center for Laboratory Animal Sciences, National Institute of Nutrition, Hyderabad, India, were injected intraperitoneally with 90 mg/kg body weight STZ dissolved in 0.1 M citrate buffer, pH 4.5 (*n* = 23). Control pups (*n* = 7) received only vehicle. The pups were weaned after 21 days and maintained on AIN-93G/M diet in individual cages throughout the experimental period.

### 2.3. Animal Care

Animal care and protocols were in accordance with and approved by the Institutional Animal Ethics Committee (IAEC). Animals were housed in individual cages in a temperature and humidity controlled room with a 12 h L : D cycle. All these animals had free access to water. Association for Research in Vision and Ophthalmology (ARVO) statement for the use of animals in ophthalmic and vision research was followed.

### 2.4. Oral Glucose Tolerance Test

Oral glucose tolerance test (OGTT) was performed at the age of two months on rats fasted for 16 h by administering glucose orally as a bolus, at a dose of 2.0 g/kg body wt. Blood samples were collected from retroorbital plexus at 0, 30, 60, and 120 min for determining plasma glucose and insulin concentrations for assessing impaired glucose tolerance (IGT) and insulin resistance.

### 2.5. Homeostasis Model Assessment (HOMA)

Insulin resistance was assessed by homeostasis model assessment HOMA as described earlier for rats [20] using the following equation: HOMA-IR = [fasting plasma glucose (mg/dL) × fasting plasma insulin (*μ*U/mL)/2,430]. To assess insulin response of these rats to a challenge of glucose load, the area under the curve (AUC) for insulin and glucose during the OGTT was computed.

### 2.6. Clinical Parameters

Plasma glucose was measured by the glucose oxidase-peroxidase (GOD-POD) kit method (Biosystems, Barcelona, Spain) and plasma insulin by RIA kit (BRIT-DAE, Mumbai, India) method as per the manufacturer's instructions. Blood glucose was measured weekly and insulin levels were measured at the age of two months and at the end of the experimental period of seven months.

### 2.7. Slit-Lamp Examination and Cataract Classification

Eyes were examined every week using a slit-lamp biomicroscope (Kowa SL-15 Portable, Japan) on dilated pupils. Initiation and progression of lenticular opacity was graded into four categories as reported earlier [21].

### 2.8. Collection of Lens and Pancreas

At the end of seven months, experiment was terminated. Pancreas and eyeballs were collected. Eyeballs were dissected using the posterior approach and lenses were frozen at −80°C until further analysis. Pancreas was collected and fixed in Bouin's solution for 24 h and then washed with 70% ethanol and stored in 70% ethanol until further analysis.

### 2.9. Immunohistochemistry of Pancreas

The tissues were later paraffin embedded and blocks were prepared using Histocentre 3 (UK). The sections were deparaffinized by incubating the sections with xylene for 10 min each for three times. Then sections were dehydrated with decreasing grades of alcohol (90%, 70%, and 50%). Antigen retrieval was done by boiling the slides in 0.1 M citrate buffer, pH 6 for 20 min. The endogenous peroxidase activity was quenched by incubating the slides in 3% H_2_O_2_ for 30 min followed by two washes with PBS up to 5 min each. To prevent nonspecific binding of the antibody, blocking was done by incubating the slides in 10% normal goat serum in PBS at room temperature for 1 h. Slides were incubated overnight at 4°C with anti-rat polyclonal insulin primary antibody raised in rabbit in PBS containing 1% normal goat serum. Slides were washed with PBS and incubated further with biotinylated anti-rabbit secondary antibody for 30 min followed by incubation with VECTASTAIN Elite ABC reagent (three-layer streptavidin-biotin peroxidase complex staining method, Vector Laboratories). Sections were developed using 3% H_2_O_2_ and diaminobenzidine tetrahydrochloride (DAB) and observed under microscope. Results were expressed in the form of average percent of insulin positive cells in the islets.

### 2.10. Lens Weight and Protein Content

A 10% homogenate was made from 4 to 5 pooled lenses in 50 mM phosphate buffer, pH 7.4. Before centrifugation, a set of aliquots were made for estimation of total protein, lipid peroxidation, and sorbitol. The remaining total homogenate was centrifuged at 10,000 ×g for 30 min at 4°C. The supernatant was referred to as the soluble fraction. The pellet was washed twice with 50 mM phosphate buffer, pH 7.4 and the wash was pooled with the supernatant. Total and soluble protein content was estimated with the Lowry method, and the percentage of soluble protein was calculated.

### 2.11. Measurement of Polyol Pathway Intermediates

The status of the polyol pathway in the eye lens of control, experimental rats was assessed by analyzing the activity of aldose reductase (AR) and sorbitol levels. The activity of AR and sorbitol levels were assayed according to the methods reported earlier [21, 22].

### 2.12. Oxidative Stress

Lens lipid peroxidation was measured as thiobarbituric acid reacting substances (TBARS) and protein carbonyl content was determined based on their reactivity with 2,4-dinitrophenylhydrazine according to reported methods [21, 22, 44]. In brief, malondialdehyde is the end product of lipid peroxidation of polyunsaturated fatty acids. Malondialdehyde was estimated by utilizing its reactivity with 2-thiobarbituric acid resulting in a pink coloured condensation product, a trimethine which was measured spectrophotometrically at 533 nm. The carbonyls react with 2,4-dinitrophenylhydrazine to form protein hydrozones, which can be detected and quantified spectrophotometrically at 365 nm.

### 2.13. Antioxidant Enzymes

The activities of antioxidant enzymes, superoxide dismutase (SOD), glutathione S-transferase (GST), and glutathione peroxidase (GPx) were assayed spectrophotometrically according to the reported methods [21, 22, 44]. The assay of SOD is based on the ability of the enzyme to inhibit the autoxidation of pyrogallol. The rate of autoxidation of pyrogallol is measured following the change in absorbance at 420 nm. GST catalyzes the conjugation of toxic electrophilic compounds with glutathione. During this assay formation of 1-chloro-2,4-dinitrobenzene-glutathione conjugate was monitored at 340 nm. GPx catalyzes the oxidation of reduced glutathione by hydrogen peroxide or lipid peroxides to oxidized glutathione (GSSG). The rate of GSSG formation, a measure of enzyme activity, was monitored coupling with the glutathione reductase reaction where NADPH oxidation was followed at 340 nm.

### 2.14. Statistical Analysis

One-way ANOVA was used for testing statistical significance between groups of data, and individual pair difference was tested by means of Duncan's multiple-range test. Heterogeneity of variance was tested by the nonparametric Mann-Whitney test. *P* < 0.05 was considered significant.

## 3. Results

### 3.1. Induction of IGT or Diabetes

After 8 weeks, experimental (STZ injected) rats were assessed for the development of hyperglycemia by estimating fasting blood glucose. nSTZ rats were subdivided into two groups depending upon their fasting plasma glucose levels. Those without fasting hyperglycemia (<110 mg/dL) were considered as prediabetic (nSTZ-PD; *n* = 16) and those with fasting hyperglycemia (>110 mg/dL) were considered as diabetic (nSTZ-D; *n* = 7). The experiment was continued for another 5 months (total 7 months) for development of cataract.

### 3.2. General Characteristics

The characteristics of control and nSTZ animals are shown in Table 1. There was an increased food intake in nSTZ-D rats when compared with control. Despite increased food intake, the body weight of nSTZ-D animals was decreased, when compared with the control rats. Though there was no significant difference in food intake of nSTZ-PD rats, the body weights of these animals were decreased, when compared with control (Table 1).

### 3.3. Blood Glucose and Insulin

**Figure 1:**
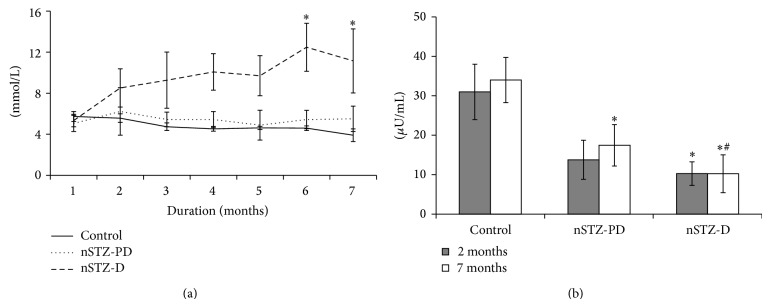
Plasma glucose and insulin levels during experiment. Fasting glucose levels (mg/dL) were estimated once in a week. Values represent mean ± SD (*n* = 7–16). The asterisk denotes that data are significantly different from control group and hash mark denotes significantly different from nSTZ-PD group.

**Figure 2:**
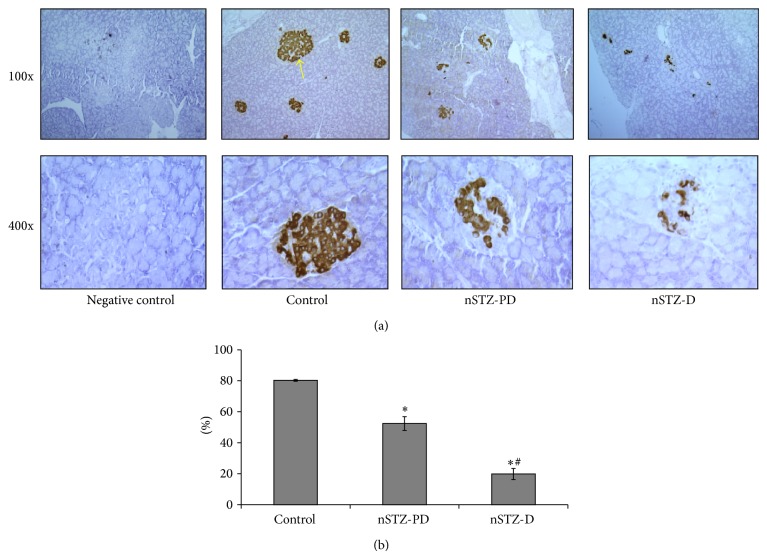
Immunohistochemistry of insulin positive cells in pancreas. Pancreatic beta cells were identified by insulin monoclonal antibody (a) and the insulin positive cells were counted by Leica Laser Microdissection microscope (b). Arrow indicates islet. The values are mentioned as percentage beta cells against total islets cells (b). Values represent mean ± SD (*n* = 3). The asterisk denotes that data are significantly different from control group and hash mark denotes significantly different from nSTZ-PD group.

Fasting blood glucose levels of nSTZ-PD as well as nSTZ-D animals were comparable at the age of one month. However, at the age of two months only 30% of the all STZ injected rats (7 out of 23) developed hyperglycemia and they continued to be in hyperglycemic state throughout the experiment. Interestingly, the remaining 70% (17) rats showed normal fasting glucose at the age of two months and they maintained the normal fasting glucose even up to the age of seven months (Figure 1). As expected a significant (*P* < 0.001) decrease in insulin levels was observed in nSTZ-D rats when compared to controls. Interestingly, in spite of significantly (*P* < 0.001) low levels of insulin, nSTZ-PD rats exhibited IGT or prediabetic characteristics (Figure 1). However, the levels of insulin in nSTZ-PD rats are higher than that of nSTZ-D rats, particularly at 7-month period. Further, these results are in accordance with percentage of insulin positive cells in pancreas of these animals as evidenced by immunohistochemistry at the end of the seven months. While there is a 50% reduction in the insulin levels as well as percentage of insulin positive cells in nSTZ-PD rats, insulin levels and insulin positive cells were about 20% in nSTZ-D rats as compared to controls (Figures 1 and 2).

### 3.4. Postprandial Glucose

Postprandial glucose is an early indicator of prediabetes and impaired glucose metabolism. Postprandial glucose was measured at two months and most of the nSTZ-PD rats had high postprandial glucose levels (227 ± 103 mg/dL) when compared with control rats (140 ± 4.16 mg/dL) indicating that majority of these rats are at IGT state.

### 3.5. Glucose Tolerance and Insulin Response

**Figure 3:**
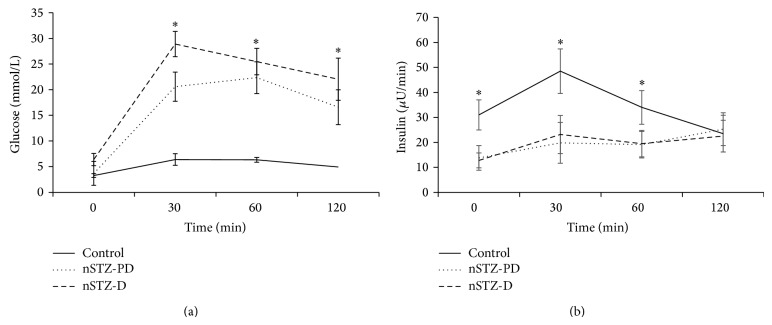
Glucose and insulin response during OGTT at two months of age. Plasma glucose (a) and insulin (b) levels in response to an oral glucose challenge in 2-month-old STZ injected (*n* = 7–16) and control (*n* = 7) SD rats following a 16 h fast. Values represent mean ± SD. The asterisk denotes that data are significantly different from control group.

Impairment of glucose tolerance in animals was assessed by OGTT conducted at the age of two months. nSTZ-PD group animals have shown the development of IGT at the age of two months as evidenced by significantly (*P* < 0.001) high levels of plasma glucose at two-hour time point during OGTT. Also, the greater area under curve for glucose in OGTT test compared to control further support the IGT characteristic of nSTZ-PD animals. However in case of nSTZ-D animals the two-hour glucose levels as well as glucose area under curve were more than that of control as a consequence of its higher fasting glucose levels (Figure 3). There is a decrease in the AUC for insulin in both nSTZ-PD and nSTZ-D rats (Figure 3).

### 3.6. Insulin Resistance and Type 2 Diabetes

Insulin insensitivity is a hallmark of T2D [45] and degree of insulin resistance was calculated by HOMA-IR. A significant (*P* < 0.01) decrease in HOMA-IR index was observed in nSTZ-PD rats when compared with controls indicating that these rats did not develop insulin resistance. As the fasting glucose levels are more in nSTZ-D rats, these rats exhibited slightly higher HOMA-IR index than nSTZ-PD animals. However this increased HOMA-IR for an nSTZ-D animal was not because of increased insulin resistance but because HOMA-IR is not a valid index during hyperglycemic stage. Further, there was a significant (*P* < 0.001) increase in glucose to insulin ratio in nSTZ groups when compared with control animals (Table 1) supporting that the nSTZ-PD group animals did not develop insulin resistance. However as indicated by glucose tolerance test nSTZ-PD animals developed IGT.

### 3.7. Slit-Lamp Examination and Cataract Progression

**Figure 4:**
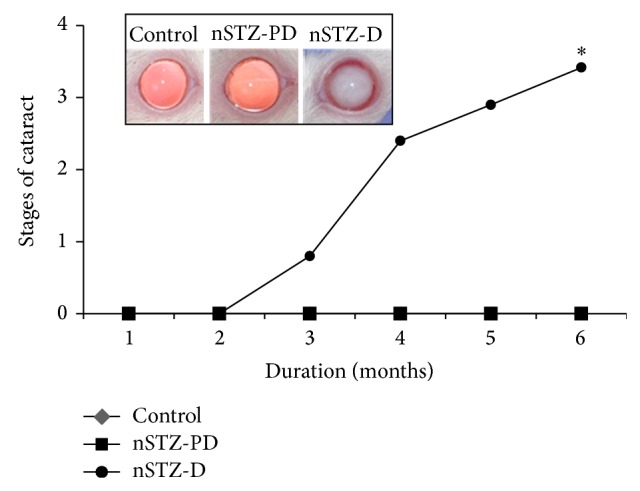
Cataract development in nSTZ induced diabetic rats. Cataract formation was monitored weekly by slit-lamp microscope and the stage of cataract was scored according to the classification described in Section 2. Stages of cataract in each group were averaged at the given time and plotted as a function of time. Representative photographs of lens from each group at the end of experiment are shown in the inset. Values represent the mean ± SD. The asterisk denotes that data are significantly different from control group.

Eyes were examined weekly for the development of lens opacity from the age of one month till the end of the experiment. As expected, the onset of cataract was observed at 9 weeks in nSTZ-D rats. However, it should be noted that the progression of cataract was very slow when compared to our previous observations with T1D rats upon injection of STZ to adult rats, where the onset of cataract was between 3 and 4 weeks after diabetes induction and developed mature cataract by 8-9 weeks [21, 22, 29]. Though nSTZ-PD rats showed IGT, they did not develop lens opacification even at the age of seven months (Figure 4).

### 3.8. Lens Weight and Protein Content

Aggregation of soluble lens proteins and increased insolubilization of proteins is one of the major biochemical alterations leading to cataractogenesis. Therefore, we determined the total, soluble and percentage of soluble protein content in the lens (Table 2). There were a significant (*P* < 0.05) reduction in lens weight in nSTZ-PD and further reduction in nSTZ-D animals when compared with controls. While soluble protein content in nSTZ-PD rats was marginally decreased, it was found to be significantly increased in nSTZ-D rats due to matured cataract as compared to control (Table 2).

### 3.9. Polyol Pathway

Activation of polyol pathway has been linked to several diabetic complications. Aldose reductase (AR), a key enzyme of polyol pathway, converts excess glucose to sorbitol, the accumulation of which is associated with many secondary complications including cataract. In the present study, while the activity of AR was marginally high in the lens of nSTZ-PD rats, the activity was significantly high in nSTZ-D rats compared to control rats (Table 3). As expected, significantly increased levels of sorbitol were observed in nSTZ-D rat lens due to hyperglycemia and high AR activity (Table 3). Though there was a marginal increase in AR activity in nSTZ-PD rats when compared with controls, there was a significant increase of sorbitol accumulation in the lens indicating that IGT state also leads to accumulation of intracellular sorbitol which further supported our previous results [20].

### 3.10. Oxidative Stress

**Figure 5:**
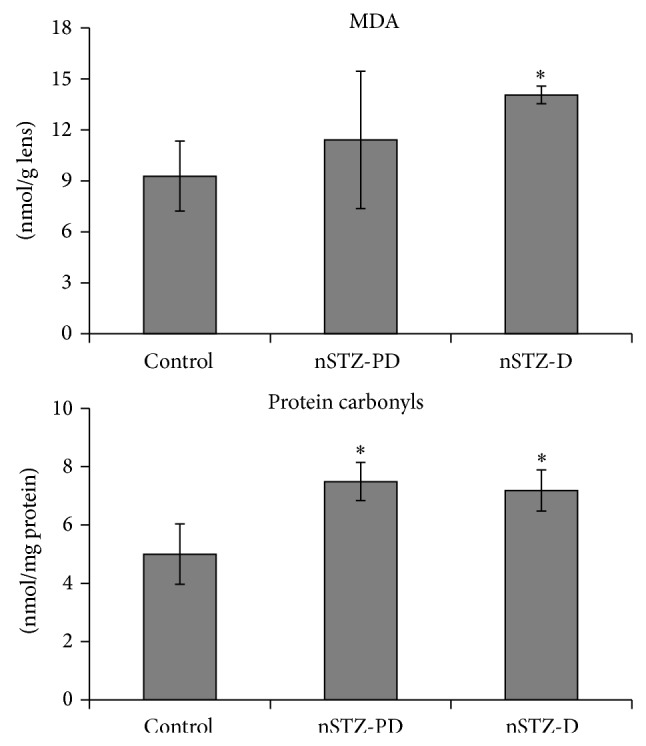
Lipid peroxidation (MDA) and protein carbonyl content in rat lens. Values are mean ± SD (*n* = 3). The asterisk denotes that data are significantly different from control group.

Oxidative stress is known to play an important role in the development of various complications during diabetic and prediabetic states. In the present study, marginal increase in lipid peroxidation was evidenced by MDA levels whereas significant increase in protein carbonyl content was observed in nSTZ-PD rats as compared to control indicating an increased oxidative stress in former group. However, as expected both these parameters were found to be significantly high in case of nSTZ-D animals (Figure 5).

### 3.11. Antioxidant Parameters

**Figure 6:**
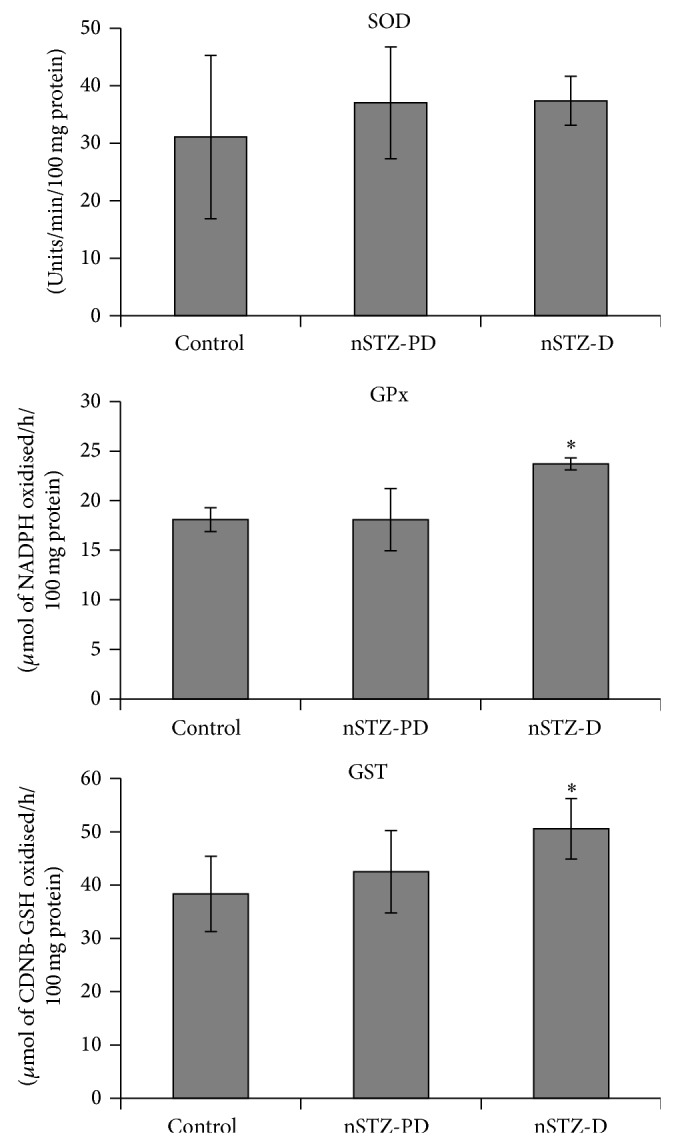
Activities of antioxidant enzymes superoxide dismutase (SOD), glutathione peroxidase (GPx), and glutathione S-transferase (GST) in lens protein. Values are mean ± SD (*n* = 3). The asterisk denotes that data are significantly different from control group.

There was a marginal increase in the activity of antioxidant enzymes such as superoxide dismutase (SOD), glutathione peroxidase (GPx), and glutathione S-transferase (GST) in nSTZ-PD rats when compared to controls (Table 3). This indicates that IGT is associated with increased oxidative stress in lens. However nSTZ-D rats exhibited significantly higher levels of GPx and GST activities (Figure 6). This indicates that oxidative stress was further enhanced by hyperglycemia.

## 4. Discussion

The progression of T2D begins with an impairment of glucose tolerance [37, 46] and is often associated with a state of insulin resistance. Several animal models have been developed to investigate T2D or IGT associated pathophysiology. nSTZ model is one of the frequently used animal models of T2D or IGT [37–40, 47] and associated complications, particularly CVD [30, 48]. In the present study, we evaluated this model for development of cataract and mechanisms responsible for cataractogenesis. According to the previous reports, STZ treated neonatal rats exhibit slightly elevated plasma glucose levels and low pancreatic insulin content at adulthood [47, 49–52]. Though in our previous study we did not observe the development of diabetic characteristics in nSTZ induced Wistar-NIN rats [20], the present study with SD rats has shown the development of IGT in majority (70%) of animals. Interestingly though all the nSTZ-PD exhibited lower fasting insulin levels, they showed normal fasting glucose throughout the experimental period.

The development of IGT, insulin resistance, or diabetic characteristics in rats depends on the STZ dose, age, duration, and strain of the rats [20, 30, 47, 48, 52–57]. Earlier it has been reported that the intraperitoneal injection of STZ (90 mg kg^−1^) in two-day-old SD rat pups developed hyperglycemia and insulin resistance at adult age [53]. However, in the present study none of the animals developed insulin resistance but majority of animals showed the development of IGT whereas the remaining developed frank hyperglycemia. These results correlated with previous reports [47, 49–52]. The major findings from this study are that though 30% of rats developed hyperglycemia around 2 months of age and continued to be in hyperglycemic state throughout the experimental period, remaining 70% of rats developed IGT and remained in IGT state till the end of the experiment without developing hyperglycemia. Indeed this situation has provided us an opportunity to investigate the impact of prediabetes or IGT on lens biochemistry* vis-a-vis* cataract development.

Thus, we have evaluated the development of cataract and associated biochemical alterations in these rats. The onset of cataract in nSTZ-D animals was observed after eight weeks of age and it matured by 24 weeks. The interesting finding of this study was that the progression of cataract in these animals was very slow when compared to our previous reports on T1D rat models [21, 22]. However, the remaining 70% animals with IGT did not develop any lenticular opacification during the study period and their lenses were apparently clear.

In general, activation of polyol pathway, nonenzymatic glycation, and oxidative stress are three major pathways that contribute significantly in the pathogenesis of diabetic complications including cataract [58]. We have also analyzed the lenses of these rats to study the possible association of IGT with lens abnormalities. As expected there was an increased activity of AR along with increased accumulation of polyols in the nSTZ-D lens indicating the activation of polyol pathway. Similarly, there were an increased lipid peroxidation, protein carbonyls, and altered SOD activity indicating the involvement of oxidative stress in lenses of nSTZ-D rats when compared with controls. However we did not observe nonenzymatic glycation as assessed by measuring AGE fluorescence in lens protein fraction (data not shown). We also evaluated the pathways in prediabetic/IGT (nSTZ-PD) rat lens. Interestingly, in accordance with our previous study [20] we observed a marginal increase in polyol and SOD indicating the activation of polyol pathway and increased oxidative stress in these animals. However, this marginal increase of polyol may not be sufficient to develop lens opacification in these nSTZ-PD rats but may be responsible for early changes in lens biochemical parameters which may subsequently lead to cataract onset and progression. However, long term studies are needed to study the actual impact of IGT on eye lens.

In conclusion, though one-fourth of nSTZ injected rats developed diabetes and subsequently cataract, majority of them developed prediabetic (IGT) state and also showed early lens biochemical changes which in due course may lead to cataractogenesis. Oxidative stress along with increased polyols was found to play a major role in the development of early lens abnormalities even in the IGT state. Hence nSTZ-PD rat model can be used to study IGT associated complications and to develop preventive strategies.

## Figures and Tables

**Table 1:** Characteristics of control, nSTZ-PD, and nSTZ-D group rats.

Parameter/group	Control (*n* = 7)	nSTZ-PD (*n* = 16)	nSTZ-D (*n* = 7)
Food intake (g/day/rat)	20.69 ± 2.21	19.08 ± 1.84	26.05 ± 4.68^#^
Body weight (g/rat)	448 ± 40.39	394 ± 47.36	364 ± 86.00^*^
HOMA-IR	0.740 ± 0.218	0.376 ± 0.243^*^	0.582 ± 0.176
Glucose AUC (mmol/h)	8.84 ± 0.85	28.40 ± 2.98^*^	36.38 ± 4.18^∗#^
Insulin AUC (*μ*mol/h)	69.25 ± 13.07	40.45 ± 9.96^*^	40.61 ± 8.98^*^
Glucose AUC/insulin AUC	0.130 ± 0.015	0.744 ± 0.269^*^	0.944 ± 0.315^*^

Values are expressed as mean ± SD. The asterisk denotes that data are significantly different from control group and the hash mark denotes that data are significantly different from nSTZ-PD group.

**Table 2:** Lens weight and protein content.

Parameter/group	Control (*n* = 7)	nSTZ-PD (*n* = 16)	nSTZ-D (*n* = 7)
Lens weight (mg/lens)	52.28 ± 4.02	48.20 ± 2.97^*^	42.77 ± 2.54^∗#^
Total protein (mg/g lens)	435 ± 72	489 ± 100	459 ± 14.55
Soluble protein (mg/g lens)	305 ± 32	329 ± 16^*^	279 ± 5.83^∗#^
Percent of soluble protein	70.11	67.28	60.78

Values are expressed as mean ± SD. The asterisk denotes that data are significantly different from control group and the hash mark denotes that data are significantly different from nSTZ-PD group.

**Table 3:** Polyol pathway parameters.

Parameter/ group	Control (*n* = 7)	nSTZ-PD (*n* = 16)	nSTZ-D (*n* = 7)
AR activity	38.20 ± 10.37	38.40 ± 10.45	42.91 ± 8.74^#^
Sorbitol	0.310 ± 0.026	0.533 ± 0.155^*^	8.738 ± 0.131^∗#^

AR activity was expressed as *μ*moles of NADPH oxidized/h/100 mg protein. Sorbitol was expressed as *μ*moles/g lens. Values are expressed as mean ± SD. The asterisk denotes that data are significantly different from control and hash mark denotes that data are significantly different from nSTZ-PD group.
